# Instar Determination for the Tomato Leafminer *Tuta absoluta* (Lepidoptera: Gelechiidae) Using the Density-Based OPTICS Clustering Algorithm

**DOI:** 10.1093/ee/nvac065

**Published:** 2022-09-06

**Authors:** Wenqian Wang, Guanli Xiao, Baoyun Yang, Jvhui Ye, Xu Zhang, Yaqiang Zheng, Bin Chen

**Affiliations:** State Key Laboratory for Conservation and Utilization of Bio-Resources in Yunnan, College of Plant Protection, Yunnan Agricultural University, Kunming 650201, China; College of Agronomy and Biotechnology, Yunnan Agricultural University, Kunming 650201, China; State Key Laboratory for Conservation and Utilization of Bio-Resources in Yunnan, College of Plant Protection, Yunnan Agricultural University, Kunming 650201, China; State Key Laboratory for Conservation and Utilization of Bio-Resources in Yunnan, College of Plant Protection, Yunnan Agricultural University, Kunming 650201, China; State Key Laboratory for Conservation and Utilization of Bio-Resources in Yunnan, College of Plant Protection, Yunnan Agricultural University, Kunming 650201, China; Resource and Utilization Research Center of Medicinal Cordyceps, Guizhou University of Traditional Chinese Medicine, Guiyang, Guizhou 550025, China; State Key Laboratory for Conservation and Utilization of Bio-Resources in Yunnan, College of Plant Protection, Yunnan Agricultural University, Kunming 650201, China

**Keywords:** OPTICS clustering algorithm, larval instar, *Tuta absoluta*, tomato leafminer

## Abstract

The tomato leafminer *Tuta absoluta* (Meyrick) is one of the most harmful pests of solanaceous crops. Its larval morphological characteristics are similar, making the distinguishing between different larval instars difficult. Accurate identification of *T. absoluta* instars is necessary either for population outbreak forecasting, or developing successful control programs. Although a clustering algorithm can be used to determine the number of larval instars, little is known regarding the use of density-based ordering points to identify the clustering structure (OPTICS) and determine the number of larvae. In this study, larval instars of 240 *T. absoluta* individuals were determined by the density-based OPTICS clustering method, based on mandible width, and head capsule width and length. To verify the feasibility of the OPTICS clustering method, we compared it with the density-based spatial clustering of applications with noise (DBSCAN) clustering algorithm, Gaussian mixture models, and *k*-means. Additionally, the instars determined by the clustering methods were verified using the Brooks–Dyar rule, Crosby rule, and linear regression model. The instars determined by the OPTICS clustering method were equal to those determined by the other types of clustering algorithms, and the instar results were consistent with the Brooks–Dyar rule, Crosby rule, frequency analysis, and logarithmic regression model. These results indicated that the OPTICS clustering method is robust for determining insect larva instar phase. Moreover, it was found that three morphological indices of *T. absoluta* can be used for determining instars of this pest in the field, which may provide important information for the management of *T. absoluta* populations.

Determination of the instar distribution of a pest population can provide important information for management because spray applications at a particular stage of pest larval development may increase the control efficacy. Thus, accurate instar determination in pests is important for forecasting the outbreak of pest insects, life table analyses, key factor analyses, and other aspects of biology (Esperk et al. 2007). The toxicity of some insecticides, as well as the efficiency larval parasitoids, and the level of expression of certain genes with instar-wise variation are instar-specific (Ramasubramanian et al. 2020). The most direct method for determining the instar number is identifying morphological features by observing the molting larvae (Puri 1925, Kiguchi and Agui 1981). However, this method is suitable for insects with significant differences in the instar morphological characteristics and short growth cycles that can be easily distinguished. For larval is that involve similar morphological characteristics, long growth periods, and spend most of their time inside leaves, stems, and fruits of plants. Thus, the instars cannot be easily distinguished.

Therefore, some rules, models, and clustering algorithms have been developed to solve this problem. Traditional instar division is based on Dyar’s rule, where a certain external morphological index of the insect body is measured (Dyar and Rhinebeck 1890). The larval development and growth rule was analyzed by the frequency distribution method to determine the age of the larvae, and the growth rule of Brooks (1886), Floater (1996), and Crosby (1973) was used to assist the inference. However, these methods are based on the fact that biological characteristics tend to have a normal distribution, the frequency density curve is bell-shaped, and the instar number is then preliminarily assumed according to the data distribution state (Mcclellan et al. 1994, Jones et al. 2005, Wang et al. 2009). If the overlap area of two normal distributions is too large, then insect larval instars cannot be determined.

To account for these problems, some cluster analyses, such as the Gaussian mixture models (GMM, Wu et al. 2013), linear discriminant method (LDA, Hunt and Chapman 2001), *k*-means clustering algorithm (Yang et al. 2018), univariate kernel density estimation (Chen and Seybold 2013), adaptive kernel smoothing method (Cen et al. 2015), adaptive bivariate kernel smoothing method (Cen et al. 2018), density-based spatial clustering of applications with noise (DBSCAN) (Zheng et al. 2019), and artificial neural networks (Carmo et al. 2020), have been proposed. DBSCAN is a typical density clustering algorithm. Compared with *k*-means clustering and Gaussian mixture models, which are generally only applicable to convex sample sets, DBSCAN can be applied to both convex and nonconvex sample sets (Hahsler et al. 2019). However, its inability to find clusters of varying densities is a notable drawback of DBSCAN, resulting from the fact that a combination of a specific neighborhood size with a single density threshold *minPts* is used to determine if a point resides in a dense neighborhood.

Ordering points to identify the clustering structure (OPTICS) is proposed based on the DBSCAN clustering algorithm to reduce the instability of the clustering results caused by the parameter settings (Ankerst et al. 1999). OPTICS borrows the core density-reachable concept from DBSCAN. However, while DBSCAN may be thought of as a clustering algorithm, searching for natural groups in data, OPTICS is an augmented ordering algorithm from which either flat or hierarchical clustering results can be derived. OPTICS introduces two additional concepts called core-distance and reachability-distance. The algorithm starts with a point and expands its neighborhood in a manner similar to that of DBSCAN, but it explores new points in the order of lowest to highest core-distance. The order in which the points are explored in addition to each point’s core- and reachability-distance is the final result of the algorithm (Hahsler et al. 2019). However, to the best of our knowledge, no study using this algorithm has focused on determining the number of insect instars in the field of entomology.

The tomato leafminer *Tuta absoluta* (Meyrick) (Lepidoptera: Gelechiidae) is an important tomato pest native to Peru, South America (Desneux et al. 2010). This insect has been found in more than 80 countries and regions in South America, Europe, Africa, Central America, and Asia (Desneux et al. 2011, Biondi et al. 2017, Campos et al. 2017, Blazhevski et al. 2018, Mansour et al. 2018, Han et al. 2019, Zhang et al. 2020). *Tuta absoluta* is considered a typical invasive species due to its capacity to develop very quickly under suitable agroecological conditions, rapidly spread in new areas, and cause economically related damage (Tropea et al. 2012, Martins et al. 2016). If no prevention and control measures are taken, then this pest can cause losses of up to 80–100% in the yield of tomato crops in recently invaded areas, and thus, it has become a major threat to global tomato production (Desneux et al. 2010). Therefore, accurate and effective prevention and control of tomato leafminer is extremely urgent.

Although previous studies found that there are four larval instars in the life cycle of *T. absoluta* (Desneux et al. 2010), because its leaf-mining and stem-, or fruit-boring behavior, and its larval morphological characteristics are similar, it is difficult to accurately determine the specific larval instar. However, because it was proven that the third instar larvae of *T. absoluta* is the most susceptible to some strains of *Beauveria bassiana* (Balsamo) (Hypocreales: Cordycipitaceae) and *Bacillus thuringiensis* (Berliner) (Bacillales: Bacillaceae), while susceptibility was lower in the second instar larvae stage (Tsoulnara and Port 2016), the determination of the specific larval instar is very important for the control this pest. Furthermore, the predatory insect *Dicyphus errans* (Wolff) (Hemiptera: Miridae) is more willing to prey on first instar larvae of *T. absoluta* (Ingegno et al. 2013). Therefore, rapid and accurate determination of the specific larval instar of the tomato leafminer is necessary for predicting its occurrence and for comprehensive prevention and control. Unless its larval instars can be accurately determined, there will be great difficulty in preventing and controlling this pest.

In this study, we used the OPTICS clustering algorithm (density-based clustering) as a new method to assess the larval instar grouping of *T. absoluta* larvae. Larval instars were measured using head capsule width and length and mandible width of the newly hatched to mature *T. absoluta* larvae. The results of the OPTICS clustering algorithm were compared with DBSCAN, another density-based clustering algorithm, as well as Gaussian mixture models (centroid-based clustering) and *k*-means clustering (distribution-based clustering). Through this work, we intend to provide a theoretical basis for studying the biological characteristics of *T. absoluta*, predicting pest outbreaks, and improving the existing integrated pest management strategy.

## Materials and Methods

### Insect Colony

The initial population of *T. absoluta* was collected in the greenhouse of Yunnan Agricultural University (102°44ʹ56ʺE, 25°07ʹ54ʺN). The experimental population was subcultured for three generations at room temperature on the Shouhe tomato.

### Measurements of *T. absoluta* Morphological Characteristics

The adults of the above populations were placed in a nylon cage with potted tomato plants (1 × 1 m, 200 mesh) and raised at room temperature. After 24 hr of oviposition, the adults were removed, and the eggs were incubated. During the entire study period (from the newly hatched larvae to mature larva pupation), 20 larvae were randomly collected daily at 9:00 a.m. The end of larval instar was identified when the mature larvae dropped from infested leaves. Larvae were rinsed from the leaves and collected into a Petri dish using a paintbrush and a 2.5-ml liquid injector and then preserved in 70% alcohol (Jones et al. 2005, Yang et al. 2018, Zheng et al. 2019). The head capsule width and length and mandible width of the larvae were measured to the nearest 0.001 cm using a Leica stereomicroscope M205FA (Leica). These three characteristics were used together in the analysis to determine the instar stage.

### OPTICS Clustering

Because OPTICS clustering was developed from the DBSCAN algorithm, we will first briefly introduce the DBSCAN algorithm. The DBSCAN algorithm is one of the most cited density-based clustering algorithms (Microsoft Academic Search 2017), and it is probably the most commonly used density-based clustering algorithm in today’s scientific community (Ester et al. 1996). The central idea behind DBSCAN and its extensions and revisions is the notion that points are assigned to the same cluster if they are density reachable from each other. The DBSCAN algorithm identifies all such clusters by systematically finding all core points and expanding each to all density-reachable points. It is not always easy to determine the appropriate values for the two parameters ϵ and *minPts*. The parameters depend on the dataset and influence each other. Detailed information regarding these clusters has been described in previous studies (Hahsler et al. 2019, Zheng et al. 2019).

The inability to find clusters of varying density is a notable drawback of DBSCAN resulting from the fact that a combination of a specific neighborhood size with a single density threshold *minPts* is used to determine if a point resides in a dense neighborhood. OPTICS requires the same ϵ and *minPts* parameters as DBSCAN. However, the ϵ parameter is theoretically unnecessary and is only used for the practical purpose of reducing the runtime complexity of the algorithm. To describe OPTICS, we introduced two additional concepts called core-distance and reachability-distance.

#### Definition 1:

*ϵ*-neighborhood*. The* ϵ-*neighborhood,*$N_{\epsilon }\left(\;p\right)$*, of a data point p is the set of points within a specified radius ϵ around p.*


$$\begin{array}{l} \left|N_{\epsilon }(\;p)\right|=\left\{q\in D\;\;\;|\;d\left(\;p,q\right)<\epsilon \right\} \end{array}$$

*where d is some distance measure and* $\epsilon \in R^{+}$ *. Note that together with* p∈D *this definition implies that point p is always part of its own ϵ-neighborhood, i.e., p ϵ always holds. The size of the neighborhood* $\left|N_{\epsilon }(\;p)\right|$ *showed as a simple unnormalized kernel density estimate around p using a uniform kernel with a bandwidth of ϵ.*

…

#### Definition 2:

Core-distance. *The core-distance of a point p*∈*D with respect to minPts and ϵ is defined as:*


$$\begin{array}{l} core-dist\;\left(\;p;\epsilon ,minPts\right)=\left\{ \begin{array}{l} UNDEFINED\\ minPts-dist\left(\;p\right) \end{array}\right.\begin{array}{l} if\left|N_{\epsilon }(\;p)\right|<minPts,and\\ otherwise \end{array} \end{array}$$

*where minPts-dist(p) is the distance from p to its minPts-*1 *nearest neighbor, i.e., the minimal radius a neighborhood of size minPts centred at and including p would have.*

#### Definition 3:

Reachability-distance. *The reachability-distance of a point*p∈D*to a point*q∈D*parameterized by ϵ and minPts is defined as:*


$$\begin{array}{l} reachability-dist\;(p;\epsilon ,minPts)=\left\{ \begin{array}{l} UNDEFINED\\ max(core-dist\left(\;p\right),d(\;p,q) \end{array}\right.\begin{array}{l} if\left|N_{\epsilon }(\;p)\right|<minPts,and\\ otherwise \end{array} \end{array}$$

The reachability-distance of a core point *p* with respect to object *q* is the smallest neighborhood radius such that *p* would be directly density-reachable from *q*. Note that the parameters, although they have the same name, work differently than those in DBSCAN. OPTICS is typically set to a very large value compared to that of DBSCAN. Therefore, OPTICS will consider additional nearest neighbors in the core-distance calculation, and *minPts* affects the smoothness of the reachability distribution, where larger values will lead to a smoother reachability distribution. This scenario needs to be considered when choosing appropriate parameters. It is worth noting that the ϵparameter is strictly used for computational reasons, and it restricts the number of points considered in the neighborhood search. It can safely be set to the maximum *k*-nearest neighbor (*k-*NN) distance, where *k* = *minPts*, and will achieve the same result as if *ϵ* were set to∞. Detailed information regarding these clusters has been described in previous studies (Hahsler et al. 2019).

The instar results for the OPTICS algorithm were assessed by the Brooks–Dyar and Crosby rules, frequency analysis, and logarithmic regression model (Dyar and Rhinebeck 1890, Crosby 1973). Detailed information regarding these procedures has been described in previous studies (Wu et al. 2013, Yang et al. 2018, Zheng et al. 2019).

### Comparative Tests

To verify that the OPTICS clustering method is appropriate, DBSCAN clustering, *k*-means clustering, and Gaussian mixture models were also used to determine instars for *T. absoluta*. Detailed information regarding DBSCAN clustering is described above. Gaussian mixture model-based clustering was conducted based on the maximum-BIC model selected using the Bayesian information criterion (BIC). The BIC for parameterized Gaussian mixture models was fitted using the EM algorithm initialized by model-based hierarchical clustering. Detailed information on Gaussian mixture models has been described in previous studies (Wu et al. 2013, Scrucca et al. 2016, Zheng et al. 2019). Detailed information on *k*-means clustering has also been described in previous studies (MacQueen 1967, Wagstaff et al. 2001, Yang et al. 2018). The instar results for the above three clustering methods were also assessed by the Brooks–Dyar and Crosby rules, frequency analysis, and logarithmic regression model (Dyar and Rhinebeck 1890, Crosby 1973).

### Statistical Analysis Software

The statistical analyses were performed in R software, version 4.1.0 (R Development Core Team, 2017). OPTICS and DBSCAN clustering were performed using the ‘dbscan’ package (Hahsler et al. 2019, Zheng et al. 2019). Gaussian mixture models were performed using the ‘mclust’ package (Scrucca et al. 2016), and *k*-means clustering was performed using the ‘Amap’ package (Lucas 2019). Linear regression equation fitting was performed using the ‘basicTrendline’ package (Mei et al. 2018). Data visualization was performed using the ‘ggpubr’, ‘ggplot2’, and ‘scatterplot3d’ packages (Ligges et al. 2002, Kassambara 2017).

## Results

### Determining *T. absoluta* Larval Instars by the OPTICS Clustering Algorithm

**Fig. 1.**
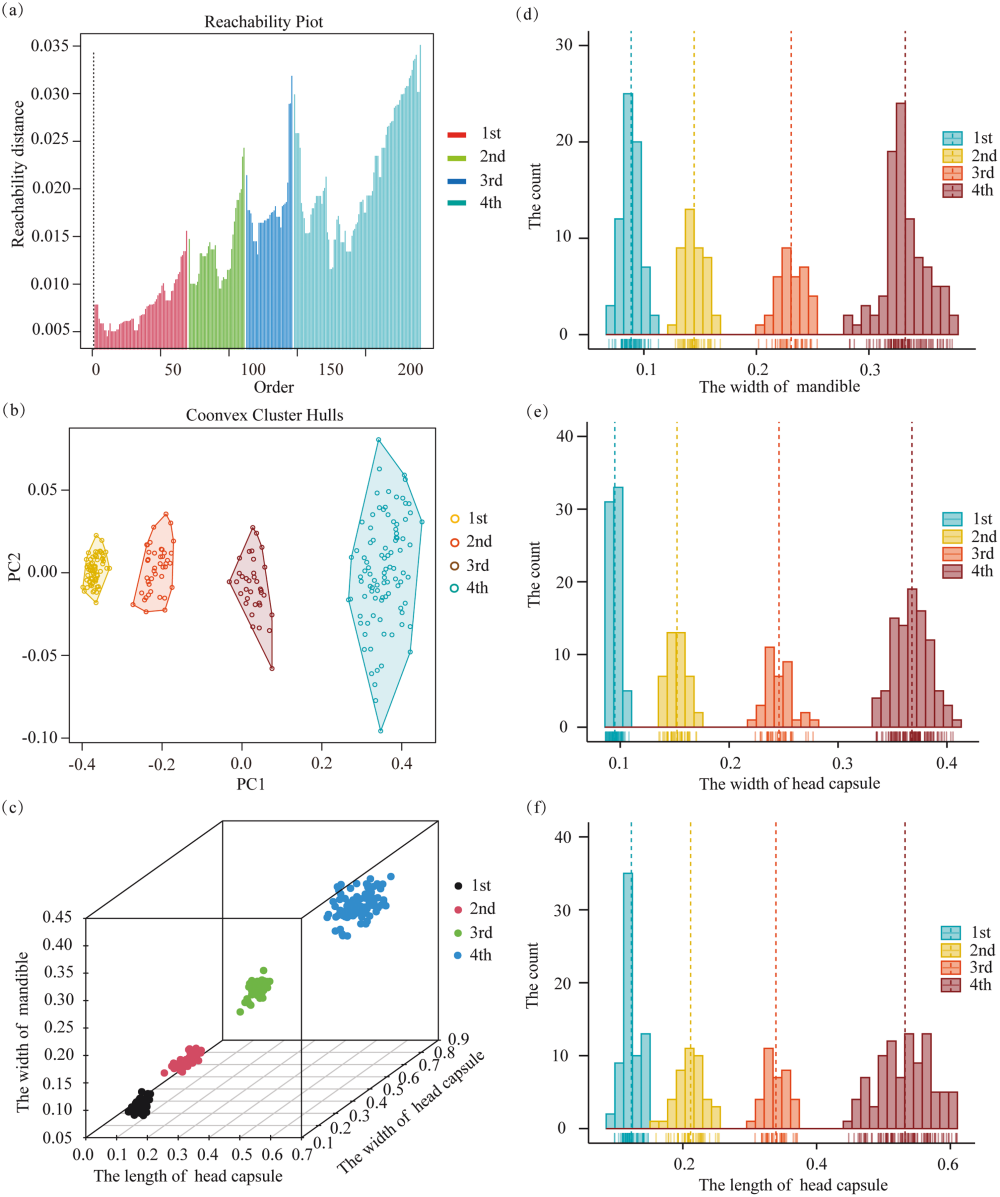
Determining the *T. absoluta* larval instar stage using the ordering points to identify the clustering structure (OPTICS) clustering algorithm. (a–c) The results for *T. absoluta* larval instars, as determined by the OPTICS clustering algorithm map using the original data; (d–f) OPTICS clustering algorithm map of size-frequency distributions. The different *T. absoluta* larval instars determined using the OPTICS clustering algorithm are annotated with different colors.

The OPTICS clustering algorithm was performed at a default *MinPts* of 5 points, and the results are shown as a reachability plot and convex hull plot (Fig. 1a and b). The OPTICS clustering algorithm was used on 240 larval individuals to correctly categorize them into one of four *T. absoluta* larval instar stages using head capsule width and length and mandible width. A total of 69, 42, 35, and 94 larvae at the first, second, third, and fourth instar phases, respectively, were recorded.

The averages of each clustering group, as determined by the OPTICS clustering algorithm, were verified using the Brooks–Dyar rule, Crosby rule, size-frequency distribution, and linear regression model of the Brooks–Dyar equation (Table 1 and Fig. 1). The Brooks–Dyar index of head capsule length and width and mandible width calculated using the OPTICS clustering algorithm ranged from 1.569 to 1.720, 1.497 to 1.601, and 1.439 to 1.632, respectively, indicating a constant rate. The Crosby index of major morphological characteristics determined by the OPTICS clustering algorithm was less than 10%, showing that the cluster determined by the OPTICS clustering algorithm was sustainable.

**Table 1.** Statistical analyses of the *T. absoluta* larval instars using four types of clustering algorithms

Cluster analysis	Variable	Instars	Larva count	Mean ± S.E. (cm)	Variation (cm)	Coefficient of variance (%)	Brooks–Dyar index	Crosby index
OPTICS clustering algorithm/DBSCA-N clustering algorithm/ Gaussian mixture models/*k*-means clustering	Head capsule length	1	69	0.123 ± 0.012	0.098–0.148	9.683		
		2	42	0.212 ± 0.021	0.160–0.254	9.997	1.720	
		3	35	0.339 ± 0.016	0.308–0.371	4.741	1.603	−0.073
		4	94	0.532 ± 0.040	0.448–0.610	7.464	1.569	−0.022
	Head capsule width	1	69	0.190 ± 0.010	0.173–0.216	5.227		
		2	42	0.304 ± 0.017	0.271–0.340	5.739	1.601	
		3	35	0.492 ± 0.025	0.448–0.554	5.093	1.616	0.010
		4	94	0.736 ± 0.032	0.670–0.811	4.410	1.497	−0.080
	Mandible width	1	69	0.089 ± 0.008	0.069–0.113	9.300		
		2	42	0.145 ± 0.010	0.128–0.168	6.716	1.632	
		3	35	0.231 ± 0.012	0.202–0.254	5.294	1.596	−0.023
		4	94	0.332 ± 0.019	0.283–0.374	5.724	1.439	−0.109

The *T. absoluta* larval instars determined by the OPTICS clustering algorithm were mapped to the size-frequency distribution (Fig. 1d–f). The clusters of all morphological characteristics fit the size-frequency distribution well, and there was no overlap in the histogram of the size-frequency distribution. We also performed principal component analysis (PCA) (Fig. 1b), and the results showed that the four clusters produced by the OPTICS clustering algorithm were completely mapped.

**Fig. 2.**
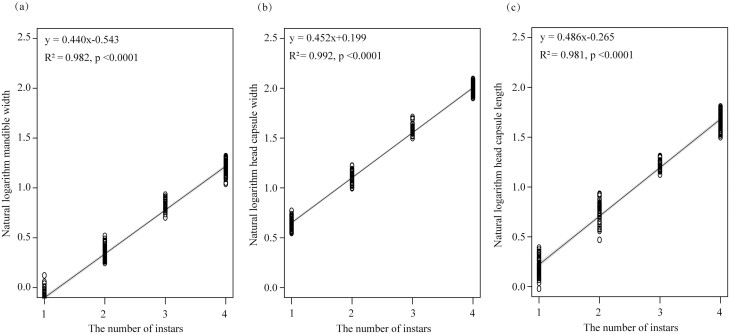
Linear regression relationship between the measurements of the morphological characteristics with 95% confidence intervals and instar number. (a) Mandible width, (b) head capsule width, and (c) head capsule length.

The log-transformed size of the morphological characteristics in each clustering group, as determined by the OPTICS clustering algorithm, was plotted using the linear regression model of the Brooks–Dyar equation (Fig. 2). The Brooks–Dyar equation for mandible width was *y*=0.440 *x*-0.543 (*R*^*2*^ = 0.981, df *=* 238, *RSS* (residual sum of squares) = 1.392, *p* < 0.0001), and the growth rate constant *e*^*b*^ was 1.553. The Brooks–Dyar equation for head capsule width was *y*=0.452 *x*+0.199 (*R*^*2*^ = 0.992, df *=* 238, *RSS*=0.657, *p* < 0.0001), and the growth rate constant *e*^*b*^ was 1.571. The Brooks–Dyar equation for head capsule length was *y*=0.486 *x*-0.265 (*R*^*2*^ = 0.981, df *=* 238, *RSS*=1.773, *p* < 0.0001), and the growth rate constant *e*^*b*^ was 1.626. Thus, the size of each morphological characteristic exponentially increased with the *T. absoluta* larval instars. The results also showed that the clusters created by the OPTICS clustering algorithm were consistent with those created by the Brooks–Dyar rule, further supporting its reliability for determining the number of *T. absoluta* larval instars.

### Comparative Tests

**Fig. 3.**
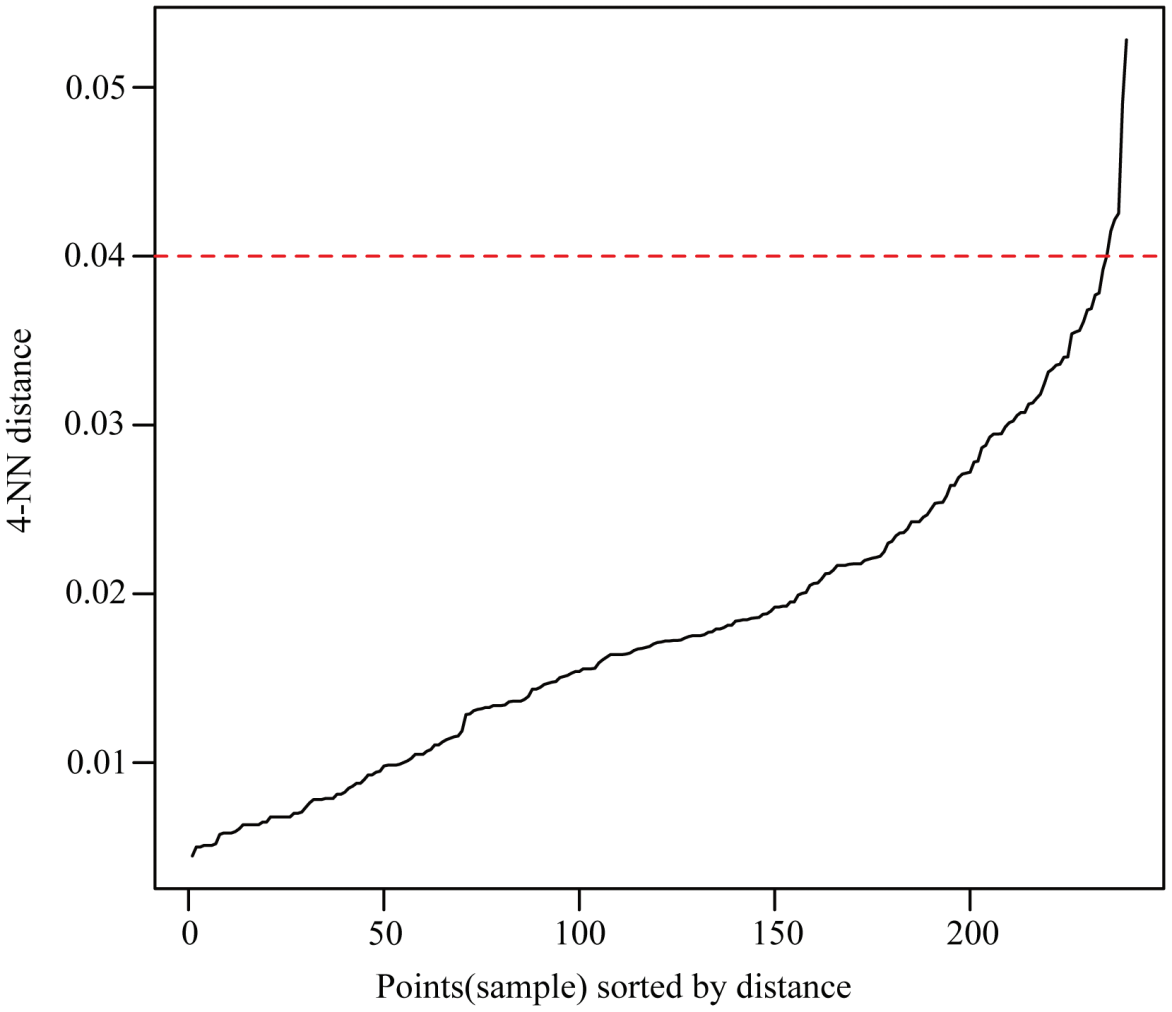
*k*-nearest neighbor (*k*-NN) distance plot (a knee—the optimal neighborhood radius eps—is visible near a 4-NN distance of 0.04).

**Fig. 4.**
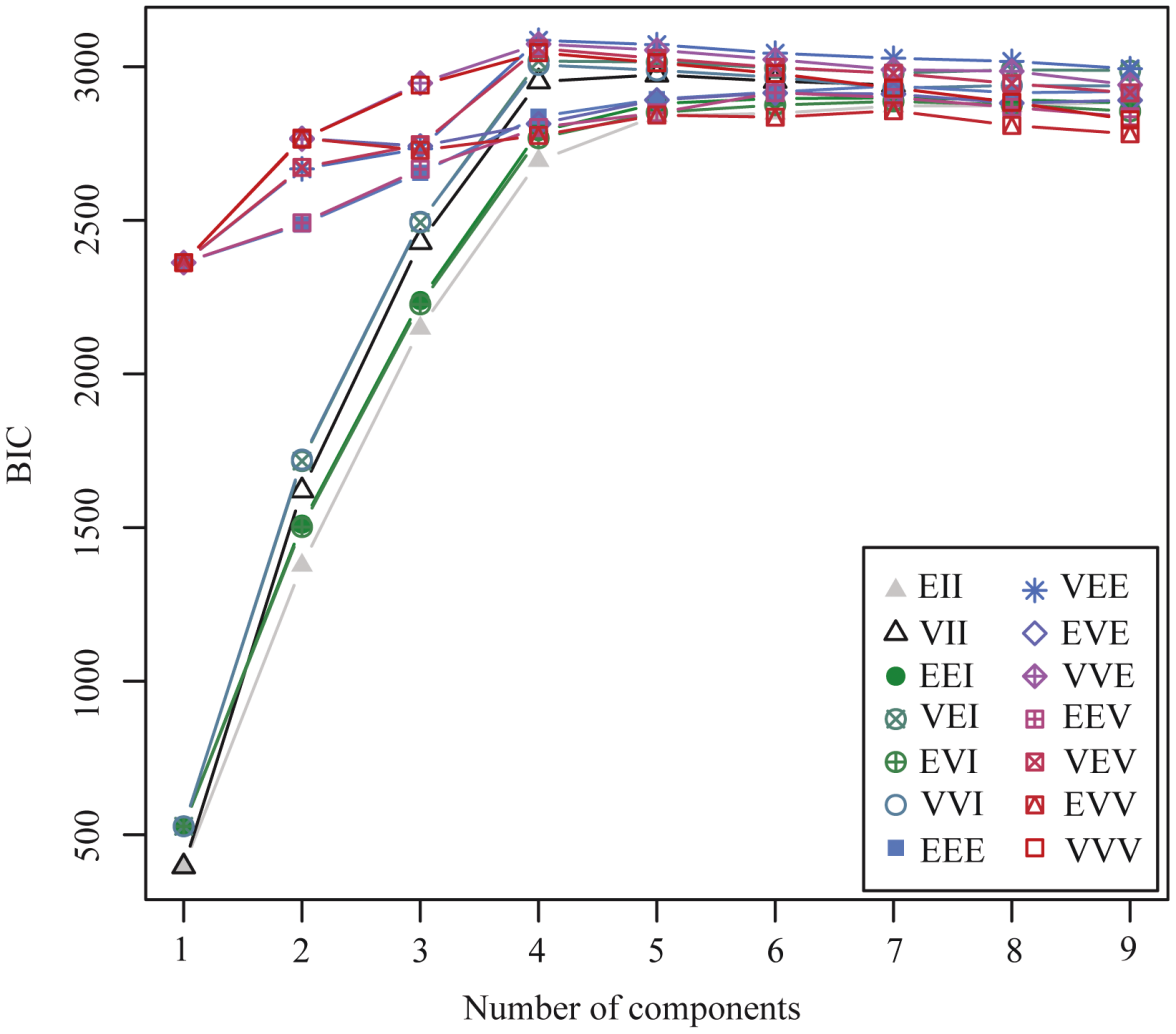
Bayesian information criterion (BIC) plot of the measurement dataset of the *T. absoluta* larval morphological characteristics. Note: ‘EII’ is spherical and has an equal volume; ‘VII’ is spherical and has an unequal volume; ‘EEI’ is diagonal and has an equal volume and shape; ‘VEI’ is diagonal and has a varying volume and equal shape; ‘EVI’ is diagonal and has a varying volume and varying shape; ‘VVI’ is diagonal and has a varying volume and shape; ‘EEE’ is ellipsoidal and has an equal volume, shape, and orientation; ‘EVE’ is ellipsoidal and has an equal volume and orientation; ‘VEE’ is ellipsoidal and has an equal shape and orientation; ‘VVE’ is ellipsoidal and has an equal orientation; ‘EEV’ is ellipsoidal and has equal volume and equal shape; ‘VEV’ is ellipsoidal and has an equal shape; ‘EVV’ is ellipsoidal and has an equal volume; and ‘VVV’ is ellipsoidal and has a varying volume, shape, and orientation. Where the maximum is taken over all of the models and numbers of components considered.

To verify that the OPTICS clustering algorithm is appropriate and scientific, DBSCAN clustering, *k*-means clustering, and Gaussian mixture model creation were performed. The same instar clusters determined by the OPTICS clustering algorithm were determined by the DBSCAN clustering, *k*-means clustering, and Gaussian mixture models. For DBSCAN clustering, the same instar clusters were obtained with a neighborhood radius eps of 0.04 and a minimum number of points (*MinPts*) of 4 (Fig. 3). According to the largest BIC value (Fig. 4), the unconstrained model with four clusters was selected, and the same instar clusters were determined by the Gaussian mixture models. For *k*-means clustering, the same instar clusters were obtained at *k* = 4.

The clusters of all morphological characteristics, as determined by DBSCAN clustering, *k*-means clustering, and the Gaussian mixture models, were the same as those determined by the OPTICS clustering algorithm and fit the Brooks–Dyar rule and Crosby rule well (Table 1). Thus, OPTICS clustering can be used for determining *T. absoluta* larval instars.

## Discussion

In this study, the density-based OPTICS clustering algorithm was used to determine the number of *T. absoluta* larval instars based on head capsule width and length and mandible width, and the results were the same as those obtained through the DBSCAN clustering algorithm (density-based clustering), Gaussian mixture models (centroid-based clustering), and *k*-means clustering (distribution-based clustering) and fit the Brooks–Dyar and Crosby rules, and size-frequency distribution well. Our results showed that *T. absoluta* larval instars were reliably and effectively determined by the OPTICS clustering algorithm and three other types of clustering algorithms. The results indicated that this density-based clustering method is a promising tool for the identification of *T. absoluta* larval instars. It will not only be the necessary raw material for forecasting but also for developing successful control programs for *T. absoluta*.

Gaussian mixture models, as distribution-based clustering has been used in determing the instars of *Blaptica dubia* (Serville) (Blattodea: Blaberidae) (Wu et al. 2013), *k*-means clustering, as centroid-based clustering has been used in determing the *Simulium quinquestriatum* (Shiraki) (Diptera: Simuliidae) (Yang et al. 2018). However, these algorithms are greatly influenced by subjective factors of users. For Gaussian mixture models, the clustering was conducted based on the model selected using the BIC, which is rather complicated. For *k*-means clustering, the *k* parameter signifies the user-specified number of clusters to find (Hahsler et al. 2019). Density-based clustering approaches have become increasingly popular due to their ability to capture clusters of arbitrary shapes, including nonconvex shapes. Although, density-based clustering of density-based spatial clustering of applications with noise (DBSCAN) clustering has been successfully used to determine the instars of *Phthorimaea operculella* (Zeller) (Lepidoptera: Gelechiidae) (Zheng et al. 2019). while this density-based clustering, two initial parameters *ϵ*, and *minPts* need to be manually set and entered by the user, and the clustering results are very sensitive to the values of these two parameters. Slightly different parameter settings will produce different clustering results and has some difficulties in distinguishing separated clusters if they are located too close to each other, even though they have different densities (Kanagala and Krishnaiah 2016). To overcome this problem, OPTICS algorithm was developed. Compared with the DBSCAN algorithm, the OPTICS algorithm addresses the defects of DBSCAN. OPTICS borrows the core density-reachable concept from DBSCAN. Although the OPTICS options also require the same two parameters as DBSCAN, the parameter *ϵ* is not necessary in theory and is only used to reduce the runtime complexity of the algorithm (Hahsler et al. 2019). In this study, our results show that the OPTICS clustering algorithm can be successfully determined *T. absoluta* instar stages. It should be noted that the same instar stages of *T. absoluta* clusters were determined by the OPTICS algorithm and the other algorithms, and these stages may be easily distinguishable for *T. absoluta* instar and more useful for dealing with complex data for insect instars. However, the advantages of the OPTICS clustering algorithm compared with those of the other three clustering methods are that it reduces the sensitivity to initial parameters, the clustering results are stable and unique, suggesting that the OPTICS clustering algorithm is a promising alternative approach for the identification of insect larval instars.

No overlapping size-frequency distributions for the morphological characteristics, head capsule width and length or mandible width were found, indicating that these three morphological characteristics can be distinguished in *T. absoluta* larval instars. Among the three parameters, the division of head capsule width was the clearest, indicating that head capsule width can be used as the most optimal index for age identification, and this phenomenon also exists at the instar division of many other insects (Flaherty et al. 2012, Chen and Seybold 2013, Thakur 2016, Zheng et al. 2019). In summary, the size of three morphological characteristics for different instar stages is specified by the OPTICS clustering algorithm. This data can be used for rapid and accurate instar division and increase the efficiency of integrated management strategies for this pest in the field.

The instar number of insect larvae exhibits intraspecific differences. Photoperiod, temperature, humidity, food quality and quantity, and feeding density are the most common factors affecting the instar number of insect larvae. In the case of insufficient food or unfavorable environmental conditions, the instar number of insect larvae will change (Esperk et al. 2007, Shintani and Ishikawa 2010, Goguen and Moreau 2013), and the instar number usually tends to increase under adverse rather than favorable conditions (Esperk et al. 2007). Therefore, to increase the accuracy of instar divisions and thus increase the effectiveness of pest control, it is necessary for future studies to combine field data to observe the differences between field- and laboratory-reared instars under different photoperiod population densities at different temperatures and under different nutritional conditions. Nevertheless, the use of the OPTICS clustering algorithm to determine *T. absoluta* larval instar stages is herein shown to be reliable and effective, which indicates that this method can also be used to determine the instar stages of other insects. In this study, the OPTICs algorithm was used to determine the size range of the different instars of *T. absoluta*, which provided basic information for the classification of larval instars. However, in the field, the insect is small, and its morphological indicators are still difficult to determine in filed for farmers even technicians in plant protection station, but the instar of larva can be determined using a microscope and manually measure the characteristics under the fewer samples in field. Moreover, these methods will be more practical in field when the development of simple measurement tools (such as designing a software to judge the age by taking photos) and the development of the algorithm into a simple program (such as determining the age by inputting measured data), it will be a further study.
